# *In Silico* Biomechanical Evaluation of WE43 Magnesium Plates for Mandibular Fracture Fixation

**DOI:** 10.3389/fbioe.2021.803103

**Published:** 2022-02-10

**Authors:** Vincenzo Orassi, Heilwig Fischer, Georg N. Duda, Max Heiland, Sara Checa, Carsten Rendenbach

**Affiliations:** ^1^ Berlin Institute of Health at Charité—Universitätsmedizin Berlin, Julius Wolff Institute, Berlin, Germany; ^2^ Berlin-Brandenburg School for Regenerative Therapies, Berlin, Germany; ^3^ Department of Oral and Maxillofacial Surgery, Charité—Universitätsmedizin Berlin, Corporate Member of Freie Universität Berlin, Humboldt-Universität zu Berlin and Berlin Institute of Health, Berlin, Germany

**Keywords:** finite element, mechanobiology, biomechanics, magnesium WE43, biodegradable, osteosynthesis

## Abstract

Titanium fixation devices are the gold standard for the treatment of mandibular fractures; however, they present serious limitations, such as non-degradability and generation of imaging artifacts. As an alternative, biodegradable magnesium alloys have lately drawn attention due to their biodegradability and biocompatibility. In addition, magnesium alloys offer a relatively high modulus of elasticity in comparison to biodegradable polymers, being a potential option to substitute titanium in highly loaded anatomical areas, such as the mandible. This study aimed to evaluate the biomechanical competence of magnesium alloy WE43 plates for mandibular fracture fixation in comparison to the clinical standard or even softer polymer solutions. A 3D finite element model of the human mandible was developed, and four different fracture scenarios were simulated, together with physiological post-operative loading and boundary conditions. In a systematic comparison, the material properties of titanium alloy Ti-6Al-4V, magnesium alloy WE43, and polylactic acid (PLA) were assigned to the fixation devices, and two different plate thicknesses were tested. No failure was predicted in the fixation devices for any of the tested materials. Moreover, the magnesium and titanium fixation devices induced a similar amount of strain within the healing regions. On the other hand, the PLA devices led to higher mechanical strains within the healing region. Plate thickness only slightly influenced the primary fixation stability. Therefore, magnesium alloy WE43 fixation devices seem to provide a suitable biomechanical environment to support mandibular fracture healing in the early stages of bone healing. Magnesium WE43 showed a biomechanical performance similar to clinically used titanium devices with the added advantages of biodegradability and radiopacity, and at the same time it showed a remarkably higher primary stability compared to PLA fixation devices, which appear to be too unstable, especially in the posterior and more loaded mandibular fracture cases.

## Introduction

The therapeutic gold standard for the treatment of simple mandibular fractures is open reduction and internal fixation with titanium miniplates and screws. Titanium alloys like Ti-6Al-4V are biocompatible, are resistant to corrosion, and provide high mechanical strength (Riviş et al., 2020). However, the use of titanium is associated with several drawbacks. The latter includes the induction of metal imaging artifacts in computed tomography, cone beam computed tomography, and magnetic resonance imaging (Radzi et al., 2014; Rendenbach et al., 2018; Demirturk Kocasarac et al., 2019), which reduces the diagnostic quality, *e*.*g*., in identifying malignancies and additional pathologies or to assess the progress of healing. Due to the rather high stiffness of such plate constructs, the undesired stress-shielding effect in load-bearing implants cannot be excluded and may impact union or induce even non-unions (Viljanen et al., 1995). Once the healing process is completed and implants are no longer functional (Rosa et al., 2016), their presence might interfere with facial growth (in adolescents), increase the infection risk, or be a source of metallic ion release (Katou et al., 1996; Armencea et al., 2019; Wang et al., 2019). Furthermore, when positioned in close proximity to the mental nerve, chronic pain and paraesthesia can result (Hachleitner et al., 2014). These considerations lead to the general strategy of plate removal in up to 70% of all cases, including both symptomatic and asymptomatic plates (Matthew and Frame, 1999; Bhatt et al., 2005; Van Bakelen et al., 2013; Sukegawa et al., 2020).

To overcome these challenges in metal plate fixation, alternative biomaterials for such implants, with more advantageous mechanical and biological features, have been considered. To date, mainly polymeric biodegradable options, such as polylactic acid (PLA) and composites thereof, have been tested in fracture fixation. PLA-based fixation devices have been proven to be effective in promoting bone healing in head and neck reconstructive surgeries (Suuronen et al., 1998; Sukegawa et al., 2016) and, generally, in minimally loaded anatomical locations (Moe and Weisman, 2001; Lee et al., 2010). However, concerns about the biomechanical reliability of PLA-based fixation systems in regions of high loadings, such as in the mandible, still remain. In fact, due to their low elastic modulus, these materials often lack sufficient primary fixation stability (Shetty et al., 1997; Buijs et al., 2006; Van Bakelen et al., 2013), and their use is generally limited to load-sharing indications in selected patients (Wang et al., 2016; On et al., 2020).

Alternatively, magnesium-based fixation systems have been long studied, thanks to their excellent biocompatibility and osteogenic properties (Witte et al., 2005), radiopacity, and a favorable elastic modulus closer to that of the human cortical bone than titanium. Nevertheless, the clinical use of magnesium in fracture fixation was not possible for many years due to its high reactivity *in vivo*, resulting in rapid hydrogen gas formation in bone and soft tissues during degradation by corrosion and, consequently, wound healing disorders and fixation failure (Witte, 2010). Recently, however, less reactive alloys, like WE43, and, more importantly, surface modification *via* plasma electrolytic oxidation (PEO) (Arrabal et al., 2008; Simchen et al., 2020; Hartjen et al., 2021; Rendenbach et al., 2021) have been introduced. In particular, PEO-coated WE43 alloy showed improved cytocompatibility, cell viability, and corrosion resistance compared to the corresponding non-coated alloys (Hartjen et al., 2021). Therefore, magnesium regained relevance for clinical use in load-bearing applications of reconstructive and trauma surgery. Several *in vivo* preclinical studies successfully observed new bone deposition around WE43 plates and screws (Schaller et al., 2016; Naujokat et al., 2017; Byun et al., 2020; Imwinkelried et al., 2020; On et al., 2020; Rendenbach et al., 2021; Torroni et al., 2021) and a preserved structural integrity in the first 12 weeks of the healing process (Marukawa et al., 2016; Levorova et al., 2018). However, to date, only Leonhardt *et al*. (Leonhardt *et al*., 2017; Leonhardt et al., 2021) reported a clinical application of magnesium-based lag screws for mandibular condylar head fracture fixation. No plate fixation of a midfacial or mandibular fracture has been performed in humans so far. Therefore, it remains unknown if WE43 fixation plates can provide sufficient mechanical primary stability for mandibular fracture healing.

This study aimed to investigate whether the fixation stability provided by magnesium alloy WE43 devices results in considerable changes in the biomechanical environment within mandibular fractures in comparison to the gold-standard titanium and alternative polylactide-based devices in the early stages of the healing process. A computational model of the human mandible was developed, and different fracture scenarios were simulated in association with variations in the design and material properties of the fixation devices. An evaluation of the biomechanical performance of biodegradable magnesium alloy WE43 *versus* traditional titanium alloy Ti-6Al-4V and biodegradable PLA was performed in terms of both assessment of implant failure risk and quantification of the biomechanical strain provided at the healing site.

## Materials and Methods

### Finite Element Models

**FIGURE 1:**
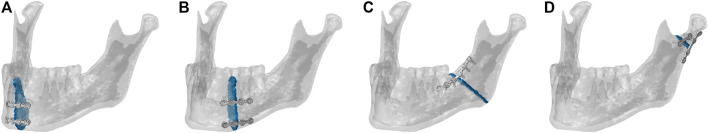
Mandibular fracture scenarios: mandibular **(A)** symphysis, **(B)** body, **(C)** angle, and **(D)** condylar neck fractures and corresponding osteosynthesis devices. The healing region is highlighted in blue.

A cone-beam computed tomography (CBCT) scan of a human skull of a 20-year-old male individual was performed in axial mode, with a voxel size of 0.4 mm³ (ProMax, Planmeca, Finland). Material segmentation of the fully dentate mandible was performed in the software Amira 6.0.1 (Zuse Institute Berlin, Germany), labeling cortical and trabecular bone tissues. Thereafter, a 3D linear tetrahedral mesh (element type C3D4) was created. The finite element model was then imported into the commercial software Abaqus/CAE v.6.18 (Dassault Systèmes Simulia Corp., United States), where the linear mesh was converted into a quadratic mesh with element type C3D10. Here four different simple fractures of approximately 1.5–1.2 mm width were simulated in the mandibular symphysis, body, angle, and condylar neck regions, respectively, as shown in Figure 1.

The fixation devices were designed in the 3D-CAD software SolidWorks 2019 (Dassault Systèmes, France) based on commercially available devices. Two different miniplate thicknesses were tested: 1-mm-thick clinically used miniplates *versus* alternative 1.5-mm-thick miniplates, in combination with simplified (without thread) monocortical 7-mm-long screws. The plates were adapted to the mandibular bone surfaces and positioned according to the four fracture scenarios (Figure 1). For the same fracture case, accurate overlapping of the 1-mm- and 1.5-mm-thick miniplates was achieved to exclude possible influences of plate positioning on the results. As shown in Figure 1, according to the principles of Champy *et al*. (1978) and the guidelines of AO Foundation ORIF (2021a) and followed by clinical advice, fracture fixation was performed with 2.0 miniplate systems. Specifically, two parallel 4-hole miniplates were used in the mandibular symphysis (AO Foundation ORIF, 2021d) and body regions (AO Foundation ORIF, 2021c). In the mandibular angle region, a 6-hole miniplate and, in the mandibular condylar neck region, a 2- and a 4-hole miniplate, both with a center space (AO Foundation ORIF, 2021b), were used. The devices were imported into Abaqus and meshed with a quadratic mesh (element type C3D10). Thereafter, tie constraints were applied between plates and screws and between screws and mandibular bone tissues.

### Material Properties

**FIGURE 2:**
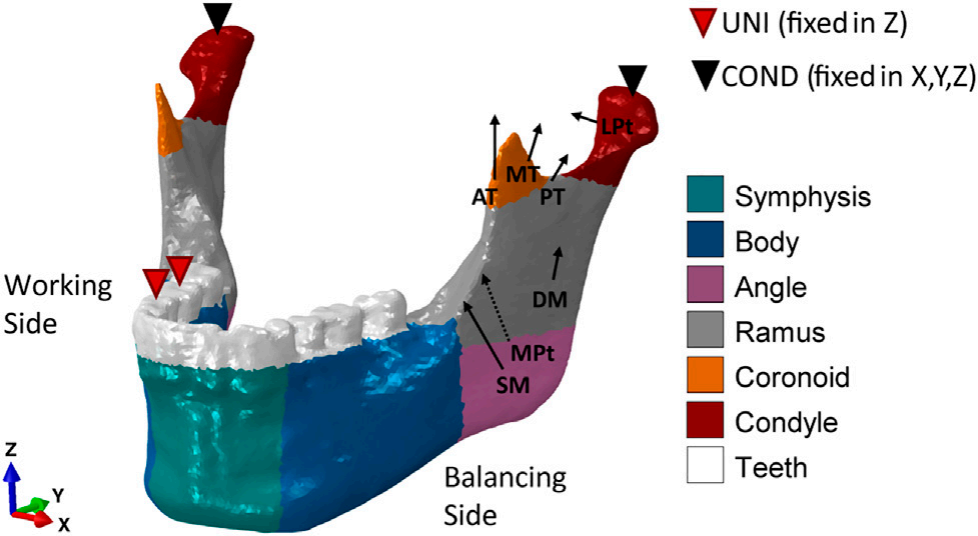
Loading and boundary conditions for the healthy human mandible during unilateral (UNI) clenching. Bite force was simulated by restraining the vertical displacement at the occlusion and both condyles in the 6 degrees of freedom. During UNI clenching, the right and the left mandibular bodies are considered as the working side and the balancing side, respectively. The cortical bone was subdivided into six regions to which different orthotropic properties were assigned (Table 1).

All material properties are reported in Table 1. Orthotropic material properties were assigned to the cortical bone, identifying six different regions according to Schwartz-Dabney and Dechow (2003) and Lovald et al. (2009) (Figure 2). Isotropic material properties were assigned to the trabecular bone (Lakatos et al., 2014), the teeth region (Lovald et al., 2009), and the fracture tissue volumes (Lovald et al., 2009), modeled as granulation tissue to simulate the early stage of bone healing.

**TABLE 1:** Orthotropic and isotropic material properties assigned to the mandibular bone tissues, fracture volume, and implants.

Material properties	Symphysis	Body	Angle	Ramus	Condyle	Coronoid process	Trabecular bone	Teeth	Granulation tissue	Ti-6Al-4V	WE43	PLA
E1	20,492	21,728	23,793	24,607	23,500	28,000	300	17,600	3	110,000	44,200	3,500
E2	16,350	17,828	19,014	18,357	17,850	17,500	300	17,600	3	110,000	44,200	3,500
E3	12,092	12,700	12,757	12,971	12,650	14,000	300	17,600	3	110,000	44,200	3,500
*ν*12	0.34	0.34	0.3	0.28	0.24	0.23	0.3	0.34	0.4	0.34	0.27	0.36
*ν*23	0.22	0.2	0.22	0.23	0.25	0.28	0.3	0.34	0.4	0.34	0.27	0.36
*ν*13	0.43	0.45	0.41	0.38	0.32	0.28	0.3	0.34	0.4	0.34	0.27	0.36
G12	6,908	7,450	7,579	7,407	7,150	7,150	—	6,567	1	41,045	17,000	1,287
G23	4,825	5,083	4,986	5,014	5,150	5,300	—	6,567	1	41,045	17,000	1,287
G13	5,317	5,533	5,493	5,386	5,500	5,750	—	6,567	1	41,045	17,000	1,287

E, elastic modulus (MPa); *ν*, Poisson’s ratio (-); G, shear modulus (MPa); direction 1, longitudinal or axial; direction 2, tangential; direction 3, transverse.

Three different material properties were assigned to the osteosynthesis devices: traditional titanium alloy Ti-6Al-4V (Lovald et al., 2009), magnesium alloy WE43 (MatWeb, 2021), and PLA (Torres et al., 2015). To define the elastic working regions of every material, the yield strength of titanium, magnesium, and PLA was assumed equal to 880 MPa (MatWeb, 2021), 162 MPa (MatWeb, 2021), and 70 MPa (Torres et al., 2015), respectively.

### Loading and Boundary Conditions

Several studies found that contralateral molar occlusion induced the highest amount of mechanical solicitation and therefore could represent a critical clenching task (Korioth et al., 1992; Lovald et al., 2009; Hijazi et al., 2016). Therefore, unilateral clenching was chosen as the only and worst clenching task to be tested in this study. Specifically, right unilateral clenching was simulated by restraining the vertical displacement of the first molar—second premolar teeth group (Figure 2). At the power stroke of mastication, the condyles were assumed locked in the glenoid fossa and therefore restrained in all 6 degrees of freedom.

Post-operative loading conditions were applied by simulating the contraction of superficial masseter (SM), deep masseter (DM), anterior temporalis (AT), medial temporalis (MT), posterior temporalis (PT), medial pterygoid (MPt), and inferior lateral pterygoid (LPt) muscles (Figure 2). The magnitude of the muscular forces was reduced to 20% of the maximum muscle forces as reported by Nelson (1986) to adapt it to post-operative muscle forces (Table 2). The muscle fiber activation patterns for unilateral clenching used in this study have been described by Nelson (1986), while the directions of the force vectors are based on Korioth *et al*. (1992).

**TABLE 2:** Force, direction cosines, and activation patterns of muscle groups for unilateral clenching in the human mandible. The muscular force components (Fx, Fy, and Fz) were obtained by multiplying the post-operative force magnitude [reduced to 20% of the maximum muscle force calculated by Nelson (1986)] by the direction cosines and the fiber activation value for both working and balancing sides (Korioth et al., 1992).

Mandibular muscle group	Maximum muscle force (N)	Post-operative muscle force (N)	Direction cosine				Fiber activation	
			X		Y	Z	Working side (right)	Balancing side (left)
			Right	Left				
Superficial masseter	190.4	38.08	−0.207	0.207	−0.419	0.884	0.72	0.60
Deep masseter	81.6	16.32	−0.546	0.546	0.358	0.758	0.72	0.60
Anterior temporalis	158.0	31.6	−0.149	0.149	−0.044	0.988	0.73	0.58
Medial temporalis	95.6	19.12	−0.222	0.222	0.500	0.837	0.66	0.67
Posterior temporalis	75.6	15.12	−0.208	0.208	0.855	0.474	0.59	0.39
Medial pterygoid	174.8	34.96	0.486	−0.486	−0.373	0.791	0.84	0.60
Lateral pterygoid	66.9	13.38	0.630	−0.630	−0.757	−0.174	0.30	0.65

XY, transverse plane; YZ, sagittal plane; XZ, coronal plane.

### Mesh Convergence Study

Four different mesh sizes were used to perform a convergence study. Mesh (A) 1,099,247 elements (finest mesh), mesh (B) 645,150, mesh (C) 288,967, and mesh (D) 168,784 (coarsest mesh) were tested. For this analysis, isotropic material properties were assigned to the bone tissues. Specifically, the elastic moduli of cortical and trabecular bone were chosen equal to 15,000 and 300 MPa, respectively, and a Poisson’s ratio of 0.3 was defined (Orassi et al., 2021). The mesh creation workflow developed in this study only generates orphan meshes (without geometry). Therefore, to avoid variability between the four meshes in the definition of the muscular attachment areas, simplified and standardized loads were applied. Specifically, forces with components Fx = 0, Fy = −50 N, and Fz = 50 N were applied at the masseter attachment on a surface of 10-mm radius during unilateral clenching. Average von Mises stresses and principal strains were calculated in the symphysis region and compared between the four cases. Mesh B, with element edges ca. 1 mm in length, showed the best compromise between accuracy and computational costs, with a relative error inferior to 5%, and was, therefore, used in this study. Eventually, the same material properties used in this study were applied to mesh B, and the results were compared to the ones obtained from the isotropic model. Comparable outcomes were observed.

### Analysis

The mechanical environment within the healing region is known to play a key role on the healing outcome. Claes and Heigele (1999) quantified strain levels lower than 15% in regions where bone formation takes place during the healing process. Therefore, the maximum and minimum principal strains within the four healing regions were calculated in all fracture scenarios for all combinations of plate thickness and material type. Specifically, the healing regions were defined as thin gaps across the bone (thickness of approximately 1.2 mm) at the mandibular symphysis, body, angle, and condyle neck (Figure 1). Strains were computed at element integration nodes to avoid possible discontinuities of the strain field at the element edges. Moreover, at each integration node, only the largest, in absolute value, between the maximum and minimum principal strains was plotted to determine, respectively, the prevalent tensile or compressive solicitation of each specific node.

Similarly, von Mises stresses within plates and screws were calculated at the integration nodes. To avoid stress singularities due to constraints between plate and screws and between screws and bone, the top 0.1% highest von Mises stress values were excluded, and the peak von Mises stresses were calculated by averaging the values in the 10 nodes with the highest stress.

## Results

In the healthy mandible, a bite force of 100 N was obtained by calculating the reaction force at the occlusion during unilateral clenching.

### von Mises Stresses Within the Fixation Devices

**FIGURE 3:**
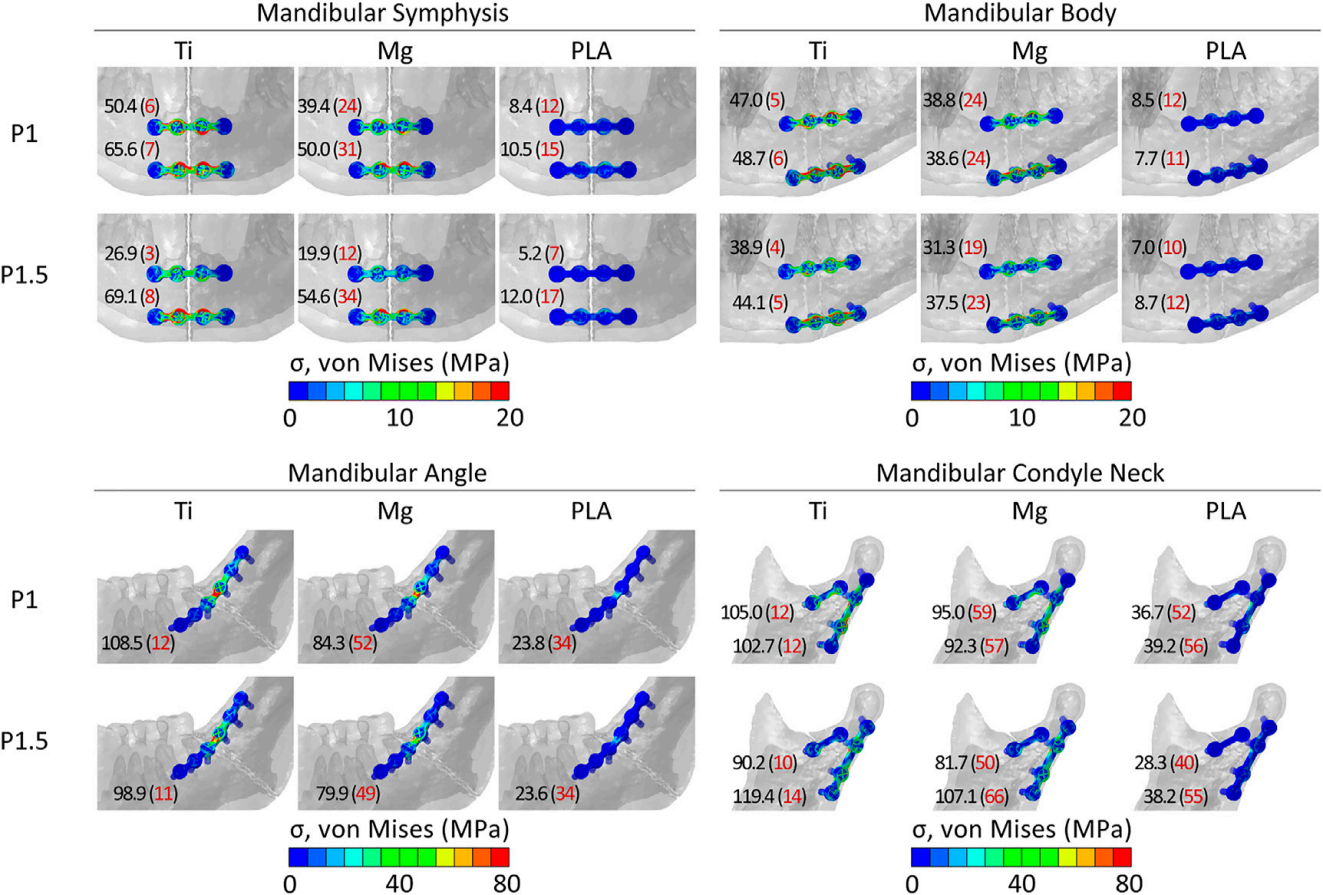
Distribution and peak values of von Mises stress within the implants in the four fracture scenarios. In red, in brackets, peak values as a percentage of the yield strength (σy) of titanium, magnesium, and PLA. σy_Ti = 880 MPa; σy_Mg = 162 MPa; σy_PLA = 70 MPa.

Concerning both plate thicknesses, the peak von Mises stresses never exceeded the yield strength of the materials. Titanium devices showed the best mechanical performance since the peak von Mises stresses ranged between 3 and 14% of titanium yield strength (880 MPa). Higher peak stress values were found in PLA and magnesium, with the latter as the closest to its yield strength but with both still working in the elastic region. Specifically, in magnesium, the peak von Mises stresses were 12–66% of magnesium yield strength (162 MPa), while in PLA, the peak von Mises stresses were 7–56% of PLA yield strength (70 MPa). Concerning the anatomical location, the highest mechanical solicitation of the fixation devices was predicted in mandibular angle and condyle neck fracture fixation (Figure 3).

### Influence of Fixation Material and Design on the Mechanical Strains Within the Healing Region

**FIGURE 4:**
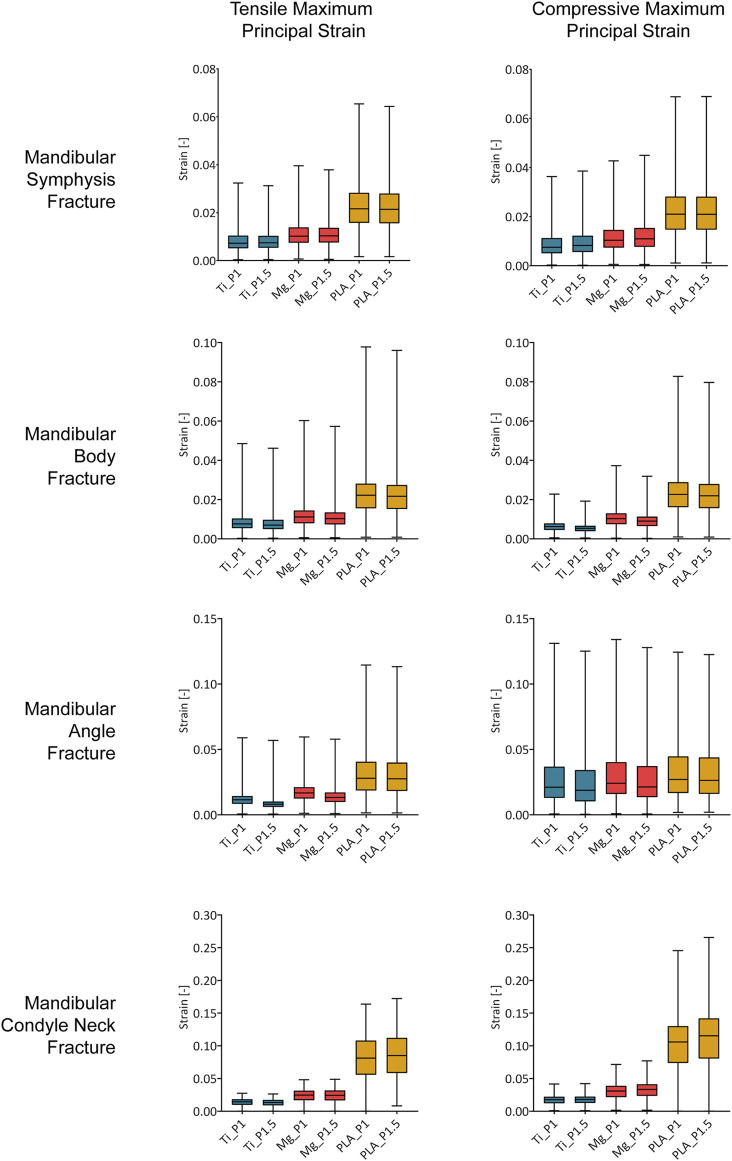
Magnitude of the tensile and compressive maximum principal strain within the healing region induced by titanium, magnesium, and PLA fixation for the two plate thicknesses in the four fracture scenarios. Ti, titanium; Mg, magnesium; PLA, polylactic acid; P1, 1-mm-thick miniplate; P1.5, 1.5-mm-thick miniplate. Each bar of the box plot contains the set of data of all nodes within the healing region, showing the frequency of the strain magnitudes.

Within the healing regions, titanium, magnesium, and PLA fixation devices induced overall tensile principal strains mainly between 0.5–1.8, 0.7–3.2, and 1.5–11.2%, respectively, and the overall compressive strains were mainly between 0.4–3.7, 0.7–4.1, and 1.5–14.2%, respectively (Figure 4).

In the case of fixation with 1-mm-thick titanium miniplates, most of the regenerating tissue volume was under strains between 0.5 and 3.7% (1st and 3rd quartiles). Similarly, 1-mm-thick magnesium miniplates induced mainly strains between 0.7 and 4.0%, while 1-mm-thick PLA miniplates induced mainly strains between 1.5 and 13.0% (Figure 4).

Small differences were predicted for an increment of miniplate thickness. Generally, the principal strains were comparable or slightly decreased using thicker plates; however, in the condyle neck fracture, both tensile and compressive strains induced by magnesium and PLA thicker plates increased. Specifically, compared to the corresponding 1-mm-thick plates, the magnesium 1.5-mm-thick plates induced tensile and compressive strains, respectively, 1 and 7% higher, while the PLA 1.5-mm-thick plates induced tensile and compressive strains, respectively, 4 and 9% higher.

### Influence of Fracture Location on the Mechanical Strains Within the Healing Region

**FIGURE 5:**
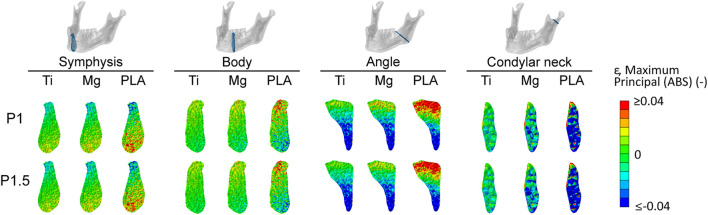
Magnitude and distribution of the maximum principal strain within the healing region induced by titanium, magnesium, and PLA fixation for the two plate thicknesses in the four fracture scenarios. Positive and negative values indicate tensile and compressive strains, respectively. Ti, titanium; Mg, magnesium; PLA, polylactic acid; P1, 1-mm-thick miniplate; P1.5, 1.5-mm-thick miniplate.

Figure 5 shows the distribution and magnitude of tensile and compressive strains within the healing region in the four fracture scenarios for all fixation designs. In the symphysis fracture, higher tensile and compressive strains were found at the inferior and crestal (top) sides, respectively, indicating mainly the bending of this region. In the body fracture, tensile and compressive strains were higher in the crestal-lingual and in the inferior-buccal sides, respectively, due to a combination of bending and torsional movements. In the angle fracture, tensile strains were mainly concentrated at the crestal side, while high compressive strains were calculated at the inferior side of the healing region due to a prevalent bending movement. In the condylar neck fracture, high tensile strains were localized at the superior border, while high compressive strains were calculated in the whole region, indicating compression and relative sliding of the fracture ends.

Regarding both mandibular symphysis and body fractures, most of the volume of the healing region (first and third quartile) (Figure 4) showed tensile and compressive strains between 0.4 and 2.9%. In the posterior fractures, the majority of the fracture volume was characterized by higher strains. Specifically, in the mandibular angle fracture, tensile values between 0.6 and 4.1% and compressive values between 1.0 and 4.5% were predicted, while in the mandibular condylar neck fracture, tensile values between 0.9 and 11.2% and compressive values between 1.2 and 14.2% were predicted.

## Discussion

In this study, the biomechanical performance of mandibular fracture fixation with magnesium WE43 plates and screws was evaluated by finite element analysis. In particular, muscle forces were reduced consistently to a post-operative bite force (Gerlach and Schwarz, 2002) to simulate the biomechanical conditions during the early stages of fracture healing. Moreover, four fracture scenarios were simulated according to the most common fractured areas in the mandible (Kaura et al., 2018) and fixated with osteosynthesis devices in traditional titanium alloy and biodegradable magnesium alloy WE43 and PLA. As compensation for the lower mechanical properties of the biodegradable materials, two miniplate thicknesses, clinically used 1-mm-thick and increased 1.5-mm-thick miniplates, were tested, both in combination with monocortical 7-mm-long screws. Subsequently, the influence of the different biomaterials and plate thicknesses on the mechanical strains within the healing region immediately after surgery (a time point known to play a role on bone healing outcome) was investigated. The results show that WE43 magnesium seems to be a suitable candidate for mandibular fracture fixation in terms of both mechanical resistance and biomechanical conditions provided at the fracture site.

The peak von Mises stresses within the fixation devices suggest that all three materials can mechanically sustain the loads in the four case scenarios without incurring a structural failure. The ratio between the peak von Mises stress and yield strength for the magnesium devices was less favorable than in the case of titanium and PLA devices; however, the predicted stresses place the WE43 magnesium alloy behavior still in the elastic region in a post-operative bite force scenario. Surprisingly, in the condylar neck region, the stress values indicate that PLA devices can withstand the masticatory loads, although they remarkably lowered the fixation stiffness.

The predicted strains in the healing regions show that metallic implants, *i*.*e*., titanium and magnesium, generally provided higher fixation stiffness compared to the polylactide implants. In particular, the difference between metallic and PLA implants was marked in the mandibular angle and condylar neck fractures, where the closeness to the muscle force application points translated into higher strains within the healing regions at both locations with PLA devices, as also found in other studies (Jung et al., 2020; Park et al., 2020). The good overlapping of the strain ranges induced by titanium and magnesium fixation devices suggests that, despite having an elastic modulus of less than half of titanium, magnesium alloy WE43 seems to ensure sufficient fixation in all tested fracture scenarios. In particular, magnesium devices also performed similarly to titanium in the more loaded posterior fractures, while PLA devices induced remarkably higher mechanical strains within the healing region, which might lead to a different healing response *in vivo* (*e*.*g*., delayed union).

Furthermore, the observed differences in the strain magnitudes between the four fracture scenarios suggest that, anteriorly (mandibular symphysis and body), bone healing happens at lower mechanical strains, while posteriorly (mandibular angle and condyles), where the muscle forces are applied, bone healing happens at higher strains. This is consistent with previous computer model predictions (Tams et al., 1999; Lovald et al., 2009; Kimsal et al., 2011). Interestingly, in this study, the calculated overall mechanical strains at the fracture sites induced by clinically used titanium devices (-4 ÷ 2%) are in the same range of strain values beneficial for bone healing in long bones (|ε|<15%) (Claes and Heigele, 1999). This suggests a similar mechano-regulation of bone healing between mandibular bone and long bones; however, this needs to be further investigated.

While changes in the implant material properties proved to highly influence fixation stiffness, according to this study, a 0.5-mm increase of plate thickness did not substantially influence either the mechanical solicitation of the devices or the biomechanical environment within the healing region. Specifically, at the mandibular body and angle fractures, a relatively small reduction of the strain level was predicted by increasing the thickness. Interestingly, at the condyle neck fracture, increased plate thickness induced slightly increased strains, using magnesium and PLA devices. The reason for this must be found in the combination of the plate thickness and material properties of the devices at this specific location. In fact, increasing the plate thickness led to a reduction of the peak von Mises stress in the 2-hole miniplate and an increase of the stresses in the 4-hole miniplate. This, in combination with the reduced stiffness of the biodegradable devices and the high torsional forces at the condylar region, determined a different and slightly higher mechanical solicitation of the healing region. To assess a possible influence of screw penetration within the bone tissues, an increased screw length (7.5 mm in length), in combination with the 1.5-mm-thick miniplates in mandibular symphysis, body, and angle fractures, was also tested, but no substantial differences in the strain values at the fracture site were induced by the longer screws (supplementary data).

The finite element model developed in this study is based on a previous finite element study of the healthy mandible (Orassi et al., 2021), which, in a maximum bite force condition, showed strain magnitudes and distribution that are in good agreement with previous computer studies (Korioth et al., 1992; Baek et al., 2012). Specifically, Korioth *et al*. (1992) found tensile and compressive strains of ca. 250 and −400 µε in the condylar neck, similar to ours, and of ca. 600 and −500 µε in the body region, slightly higher than the ones predicted in this study (400 and −300 µε). However, as shown by Baek *et al*. (2012), a high variability (>100%) of mandibular deformation can be expected between mandibles with different morphological features. In addition, in this study, in the mandibular body fracture, fixated with two parallel, 1-mm-thick titanium miniplates and a bite force of 100 N (20% of the maximum bite force), average maximum strain values of ca. 0.6–0.8% and a peak von Mises stress of 47–49 MPa were predicted. This is in agreement with the results found by Lovald *et al*. (2009), who simulated a mandibular body fracture fixated with two parallel titanium miniplates with a bite force of 320 N (60% of the maximum bite force). They observed average first principal strain values within the healing regions of ca. 1.7% and a peak von Mises stress of 87 MPa within the plates. Therefore, our results are consistent with the analysis performed by Lovald *et al*. (2009), considering that, in this study, a third of their bite force was used and small differences might exist in terms of the mandibular morphology and plate design and positioning.

This study presents several limitations—for example, fixation device material properties were defined as isotropic, homogeneous, and elastic. PLA viscoelastic behavior was not taken into account; however, cycling loading during mastication might contribute to increasing the deformation of the PLA fixation devices cycle after cycle (Pepelnjak et al., 2020), thus further reducing the primary stability at the fracture site. This implies that, in reality, PLA might provide less stability than the one predicted in our study. Another limitation of this study is the use of tie constraints for screw–bone interface modeling, which does not allow the relative motion between screws and bone that might occur in reality. However, this limitation is unlikely to influence our findings since, as also shown in another study (MacLeod et al., 2012), only the local mechanical environment in the surroundings of the screws is affected, while the global load–displacement behavior of the model remains unvaried. Moreover, the analysis did not take into account the WE43 alloy and PLA degradation process. PEO-coated magnesium WE43 has been shown to maintain up to ca. 80% of its volume at 6 months after implantation (Schaller et al., 2016), and PLA-based devices can preserve up to 70% of their initial mechanical strength after 36 weeks (Wang et al., 2016). Complete bone healing in mandibular fractures can take up to 6 months; however, functional loading is progressively restored starting from the fourth week after surgery (Friedman, 2014). Therefore, it is expected that material degradation does not highly interfere with the healing process, and it should not influence the conclusions drawn in this study due to slow degradation rates for both materials (Yerit et al., 2005; Rendenbach et al., 2021); however, this aspect remains to be further investigated.

In summary, this study aimed to investigate the biomechanical performance of magnesium WE43 alloy in mandibular fracture fixation in contrast with both clinically used titanium and softer biodegradable PLA devices. From a mechanical point of view, magnesium WE43 alloy was predicted to provide sufficient primary stiffness at the fracture site to support mandibular bone healing. The effect of plate and screw resorption on the healing process should be addressed in future studies.

## Data Availability

The datasets presented in this article are not readily available because they present some patented elements. Requests to access the datasets should be directed to SC, sara.checa@bih-charite.de.
