# Supplementary material for: In Silico Biomechanical Evaluation of WE43 Magnesium Plates for Mandibular Fracture Fixation
Source: Front Bioeng Biotechnol. 2022 Feb 10;9:803103. doi: 10.3389/fbioe.2021.803103 (PMC8866862; doi:10.3389/fbioe.2021.803103)

Supplementary Material

Supplementary Figure. Magnitude and distribution of the tensile and compressive maximum principal strain (ABS) within the healing region induced by 1.5mm-thick miniplates in combination with standard 7 mm- and alternative 7.5 mm-long screws, for mandibular symphysis, body, and angle fractures, for the three materials. The condyle neck fracture was not considered since the 7 mm-long screws already pass through the whole condylar neck. Ti: titanium; Mg: magnesium; PLA: polylactic acid; P1.5: 1.5 mm–thick miniplate; S7: 7 mm-long screw; S7.5: 7.5 mm-long screw.

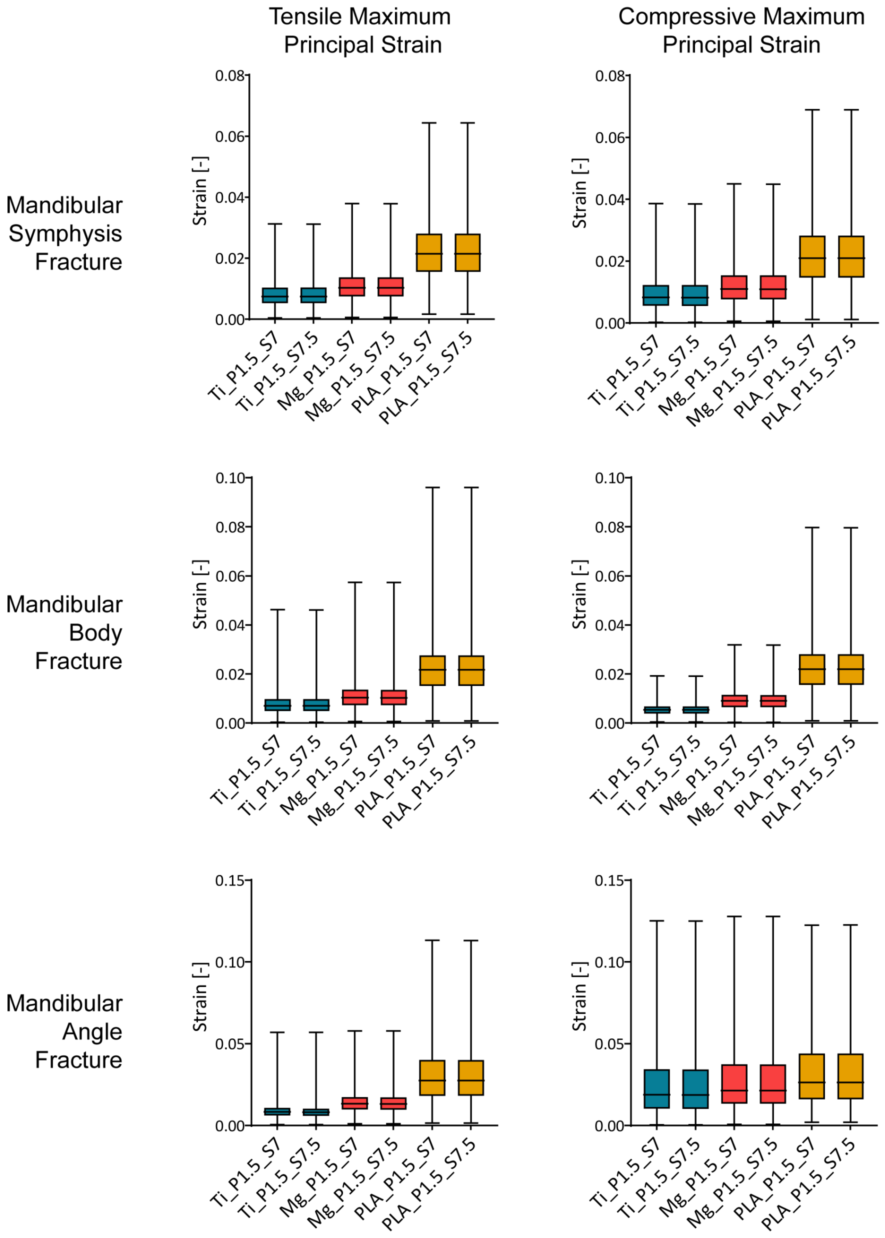

Supplement: Supplementary file 1 [file Image1.pdf]
